# Prevalence of Self-Medication Among the Elderly in Kermanshah-Iran

**DOI:** 10.5539/gjhs.v7n2p360

**Published:** 2015-01-13

**Authors:** Faranak Jafari, Alireza Khatony, Elham Rahmani

**Affiliations:** 1 Kermanshah School of Nursing and Midwifery, Kermanshah University of Medical Sciences, Kermanshah, Iran; 2 Social Development and Health Promotion Research Center, Kermanshah University of Medical Sciences, Kermanshah, Iran

**Keywords:** elderly, prevalence, reasons, self-medication

## Abstract

**Introduction:**

Self-medication is consumption of one or several medications without the physician’s prescription. Given the risks of self-medication, this study was carried out to assess the prevalence of self-medication and its related factors among the elderly in Kermanshah-Iran

**Method:**

In this descriptive cross-sectional study, 272 elderly visiting the private offices in Kermanshah were selected through convenience sampling method. The instrument for data collection was a researcher made self-medication questionnaire. Data were analyzed using descriptive and analytic statistical methods (Chi-Square and Fisher exact test).

**Results:**

The prevalence of self-medication was 83%. The most common reasons for self-medication were certainty of its safety (93%), prior consumption of the drug (87.6%), busy offices of physicians (82%), non-seriousness of the illness (77.8%) and prior experience of the disease (73%). The most common drugs used for self-medication were analgesics (92%), cold drugs (74%), vitamins (61%), digestive drugs (54%) and antibiotics (43%). There was a significant correlation between self-medication and gender (p=0.001), education level (p=008), drug information (p=0.01), marital status (p=0.002), and medical insurance (p=0.001) variables.

**Conclusion:**

considering the relatively high rates of self-medication among the elderly as well as its side effects, designing and performing educational programs are suggested for the elderly people.

## 1. Introduction

Self-medication is a serious social-health and economic problem all over the world including Iran. Self-medication means consumption of one or several drugs not prescribed by a doctor and not controlled by medical health organizations. It includes using herbal or chemical medications, previously prescribed medicine for similar cases, extra medicine at home, or not using medicine completely (Karimy et al., 2011).

Studies in some countries including Iran show a high rate of self-medication and unreasonable medicine intake, where the prevalence of self-medication in Ethiopia, Nigeria, and Nepal have been reported to be 43.2, 73 and 59 per cent, respectively (Gutem et al., 2011; Bamgboye et al., 2006; Shankar et al.,2003; Sarahroodi et al., 2012). On the other hand, the prevalence of self-medication in Iran is estimated to be three times more than the world’s average rate. It is estimated that 83.3% of Iranians use medicine on their will (Karimy et al., 2011; Sarahroodi et al., 2012).

Among all groups, the elderly tend to use higher amounts of non-prescribed medicine because they suffer more from illnesses (Sarahroodi et al., 2012). Changes related to age in metabolism and repelling medication (pharmacokinetic) as well as the effect of medicine on body (pharmacodynamic) lead to changes in body reaction to medicine among the elderly. Pharmacokinetic changes may result in congestion and aggravation of medicine effects, increasing the side effects and medicine interference (Sarahroodi et al., 2012; Vali et al., 2011). About 13% of the elderly in the US are hospitalized because of medication problems or medicine poisoning, which has resulted in 106000 deaths and has imposed 58 billion dollars expense on the U.S healthcare system (Fick et al., 2003). Based on the results of a study that was done in Iran, the medical expense of an elderly under insurance is 218 % more than a non-elderly (Nokohan et al., 2012). In addition, the prevalence of self-medication among the elderly in various cities of Iran is relatively high (Karimy et al., 2011; Sarahroodi et al., 2012; Davati et al., 2007). Considering the population patterns in Iran, the elderly have been taken into account more seriously, as their population in a census (7.3 %) in 2006 increased to 8.3% in 2011 (Vali et al., 2011; Statistic Center of Iran, 2012).

Therefore, given the increasing number of the elderly in society, side effects of using non-prescribed medicine in this age group, cultural patterns and lack of related studies in Kermanshah, the present study was conducted to investigate self-medication practice and its related factors among the elderly living in Kermanshah in 2012.

## 2. Methods

The present research was a descriptive cross-sectional study which investigated the elderly people living in Kermanshah in 2012. Among elderly who were referred to the private clinics 272 samples were selected based on the previous study (Sarahroodi et al., 2012) and using sample size equation for estimating an equation with confidence of 0.95% and accuracy of 5%. Researcher selected the patients. Based on various social-economic conditions in different districts of Kermanshah, the samples were selected in 4 different districts of Kermanshah, north, south, east and west. 68 elders, visiting private offices, were chosen from each district using convenience sampling method. The inclusion criteria into the study included being over 60, lack of hearing problem In order to participate in the interview and answer questions, lack of mental problems like Alzheimer, lack of severe physical problems like cancer, and interest to participate in the study. Data were gathered by a two-part researcher made questionnaire. The first part of the questionnaire included 11 questions about demographic characteristics such as age, gender, education, marital states, occupation, insurance, drug preservation at home, self-medication during last three months, drug information, and type of medicine used during the last three months. The second part with 11 statements was related to self-medication practice among the elderly.

Content validity was used to determine the validity of questionnaire. To this end, the questionnaire was reviewed by 12 faculty members and was revised based on their suggestions. The reliability of questionnaire was checked and confirmed by Cronbach’s alpha (0.82).

For data collection, after obtaining permission from the Vice Chancellor for Research at Kermanshah University of Medical Sciences, the researcher referred to private clinics in the four regions of the city and performed the sampling. Initially, the purpose of the study was explained to the subjects and they were assured about the confidentiality of their personal information and responses to questions. Finally, inform consent was taken from the participants. The questionnaires were completed by the researcher based on the interviews. The interviews were conducted in the private clinics.

Data were analyzed by SPSS software version 16. Descriptive statistics (frequency, mean and standard deviation) and analytical statistics (Chi-Square and Fisher exact test) were used to analyze the obtained data. The significance level was set at 0.05.

## 3. Results

From 272 patients taking part in the study, 147 persons (54%) were male. The mean age and standard deviation of the samples was 64±5.22. 63% (171) were literate and 55% (150) were single. 63% (171) had no insurance and 93% (253) had no drug information. 86% (234) kept medicine at home and 83% (226) reported self-medication (Table 1).

**Table 1:** Socio-demographic characteristic of respondents by self-medication

Characteristics	groups	Self-medication	Number (%)	*pv*
**Gender**	Male	yes	111(75.5)	0.001
		no	36(24.5)	
	Female	yes	115(92)	
		no	10(8)	
**Marital states**	Single	yes	134(89.3)	0.002
		no	16(10.7)	
	Married	yes	92(75.4)	
		no	30(24.6)	
**Educational level**	Elementary or illiterate	yes	76(75)	0.008
		no	25(25)	
	High school/college	yes	150(87.7)	
		no	21(12.3)	
**Insurance**	Yes	yes	65(64.6)	0.001
		no	36(35.6)	
	No	yes	161(94.2)	
		no	10(5.8)	
**Drug information**	Yes	yes	13(68.4)	0.01*
		no	6(31.6)	
	No	yes	213(84.2)	
		no	40(15.8)	

* Fisher exact test.

The results showed that 83 % of elderly, practiced self-medication. The most common drugs used for self-medication by the elderly were Analgesics (92%), cold tablets (74%), and vitamins (61%). The most common reasons reported by the respondents for self-medication were certainty of drug safety (93%), and previous consumption of the medicine followed by improvement (87.6%) (Table 2).

**Table 2:** Reasons of self-medication by the elderly

Reasons of self-medication	Number (%)
Prior experience about the drug	168(87.6)
No medical insurance	128(55)
Advice by friends, family and neighbors	146(64.6)
Certainty of its safety	212(93)
Availability in drugstores	21(9.2)
Saving money	103(45.5)
Saving time	187(82)
Lack of trust in doctors for diagnosis and treatment	17(7.5)
Inadequate time to attend the doctor’s office	37(16.3)
Non-seriousness of the illness	176(77.8)
Prior experience about the illness	165(73)

The results showed that self-medication was different between the male and female elderly so that women were significantly more likely than men to follow self-medication (p=0.001).

The results showed that the prevalence of self-medication was different between single and married elderly so that the single elderly were significantly more likely than the married elderly to perform self-medication (*p*=0.002).

The results showed that the rate of self-medication in individuals with diploma and above was greater than in individuals with primary education and those with no education (p=0.008).

Further, the Results showed that the prevalence of self-medication in the elderly without medical insurance was significantly higher than in those with medical insurance (p=0.001).

Moreover, the results showed that the prevalence of self-medication among the elderly without drug information was significantly higher than in the elderly with drug information (p=0.001) (Table 1).

## 4. Discussion

The findings of the present study showed that more than two third of the elderly had a history of self-medication which was higher than the rates reported in similar studies conducted in other country such as Ethiopia (43.2%), and Nepal (59%) (Gutem et al., 2011; Shankar et al., 2003). However, it was nearly similar to the results of studies in Malaysia (77.6%) and Chile (75%) (Ali et al., 2012; Fuentes et al., 2008). The differences reported about self-medication in different countries may depend on the variation in socioeconomic profiles and demographic characteristics of samples.

The most frequently consumed drugs by the elderly were analgesics, cold medicine, vitamins and digestive drugs, which are in line with the results of studies such as Davati et al. (Iran), Henry James et al. (Bahrain), Loyal et al. (Brazil), and Linjakumpu et al. (Philippines) (Davati et al., 2007; James et al., 2006; Loyola et.al, 2005; Linjakumpu et al., 2002). The high consumption of the above mentioned medicines may be because these medications are considered as over-the-counter drugs and are easily available to patients. On the other hand, prior experience about the drug may lead to increasing requests for self-medication.

The present study indicated that 86% of the elderly preserved medicine at home. This was reported to be 72% in Davati et al., 85.3% in Sarahroodi et al., 49.9% in Karimi et al. and 61% in Tajik et al.’s studies (Karimy et al., 2011; Sarahroodi et al., 2012; Davati et al., 2007; Tajik et al., 2014). Keeping medicine at home is an important issue to be considered which, in addition to increasing the possibility of self-medication, creates such concerns as expiry date, mistakes in proper consumption and availability for others.

Certainty of the safety of self-medication and prior experience about the drug were the main reasons of self-medication from the viewpoint of the elderly. Similar studies on elderly also introduced prior experience about the drug and improvement of symptoms to be the main reasons of self-medication (Karimy et al., 2011; Sarahroodi et al., 2012; Davati et al., 2007). Considering this wrong attitude and belief among the elderly, certain measures such as supervision over selling over-the-counter medicines and establishment of pharmaceutical consulting unit in pharmacies are required to be taken in order to inform the elderly about the side effects of self-medication and to change their false attitude.

The present study also revealed that self-medication was significantly higher in women than in men, which is in agreement with the results of some studies (Sarahroodi et al., 2012; Davati, et al., 2007; James et al., 2006; Olivveira et al., 2014; Abahussain et al., 2005). More self-medication practice among women may be due to the fact that they usually seek medical services more frequently than men (Ali et al., 2012).

The rate of healing in women may be due to the fact that women higher than men make use of medical services. Moreover, the findings of the present study suggested that self-medication was significantly higher among the singles than the married elderly, which is in line with the results of the studies carried out by Davati et al. and Sharifirad et al. (Sarahroodi et al., 2012; Davati et al., 2007). The authors tend to think that the support for the single elderly is lower than for the married elderly, which consequently leads to more diseases and self-medication in the single elderly.

Furthermore, the results suggested that self-medication was significantly higher among the literate elderly with secondary education and above compared with those with primary education and illiterate ones. Greater level of self- medication among the educated elderly was reported in the other studies (Karimy et al., 2011; Sarahroodi et al., 2012; Fuentes et al., 2008; Tajik et al., 2014). It seems that the educated elderly believe that they can get all needed information from medicine brochures or the previous prescriptions; therefore, they diagnose their sickness and follow self-medication using the same medications.

The results also showed that self-medication among those with drug information was significantly higher than those with no drug information, which corresponds to the results of the study by Fuentes et al. (Fuentes et al., 2008). It seems that lack of knowledge about the side effects and complications of drugs increased self-medication; however, it was less among those with information about the side effects of drugs.

In addition, the present study indicated that the elderly with no medical insurance had significantly higher levels of self-medication in comparison with those with medical insurance. This was confirmed by the some studies (Amani et al., 2011; Tajic et al., 2014); however, Sarahroodi et al. (2012) reported no statistically significant difference between self-medication and having or not having medical insurance (Sarahroodi et al., 2012). It seems that those with medical insurance tend to attend private offices more than those with no medical insurance possibly because of high physician fee.

The present study faced several limitations. The participants in this study were only the patients referring to a doctor’s office. Therefore, the results cannot be generalized to all the elderly living in Kermanshah and Iran. In this study, data were collected using interview, which may affect the accuracy of the results. Since the study was conducted only on the elderly visiting outpatient clinics, the authors suggest similar studies on the elderly across the city to provide a better analysis of the self-medication practice among the elderly. Finally, the authors recommend further research about the effect of educational interventions on self-medication in the elderly.

## 5. Conclusion

The prevalence of self-medication among the elderly was relatively high. The most common reasons given by the elderly for self-medication were certainty of its safety, prior experience about the drug, saving the time, non-seriousness of the illnesses, and prior experience about the illness. Analgesics, cold drugs, vitamins, digestive drugs and antibiotics were the most commonly reported types of medications for self-medications. Given the high prevalence of self-medication, it seems necessary to design and implement educational programs to increase the perceived risk of self-medication.
